# Non-Coding RNAs in IGF-1R Signaling Regulation: The Underlying Pathophysiological Link between Diabetes and Cancer

**DOI:** 10.3390/cells8121638

**Published:** 2019-12-14

**Authors:** Baoqing Chen, Junyan Li, Dongmei Chi, Iman Sahnoune, Steliana Calin, Leonard Girnita, George A. Calin

**Affiliations:** 1 Department of Radiation Oncology, State Key Laboratory of Oncology in South China, Collaborative Innovation Center for Cancer Medicine, Sun Yat-sen University Cancer Center, Guangzhou 510060, China; lijy1@sysucc.org.cn; 2 Department of Experimental Therapeutics, The University of Texas MD Anderson Cancer Center, Houston, TX 77054, USA; ISahnoune@mdanderson.org; 3 Department of Anesthesiology, State Key Laboratory of Oncology in South China, Collaborative Innovation Center for Cancer Medicine, Sun Yat-sen University Cancer Center, Guangzhou 510060, China; chidm@sysucc.org.cn; 4 Department of Hematopathology, The University of Texas MD Anderson Cancer Center, Houston, TX 77054, USA; steliana.calin@gmail.com; 5 Department of Oncology-Pathology, Cellular and Molecular Tumor Pathology, Karolinska Institute, 17164 Stockholm, Sweden; leonard.girnita@ki.se; 6 Center for RNA Interference and Non-Coding RNAs, The University of Texas MD Anderson Cancer Center, Houston, TX 77054, USA

**Keywords:** micoRNAs, lncRNAs, IGF-1R, cancer, diabetes

## Abstract

The intricate molecular network shared between diabetes mellitus (DM) and cancer has been broadly understood. DM has been associated with several hormone-dependent malignancies, including breast, pancreatic, and colorectal cancer (CRC). Insulin resistance, hyperglycemia, and inflammation are the main pathophysiological mechanisms linking DM to cancer. Non-coding RNAs (ncRNAs), particularly microRNAs (miRNAs) and long non-coding RNAs (lncRNAs), are widely appreciated as pervasive regulators of gene expression, governing the evolution of metabolic disorders, including DM and cancer. The ways ncRNAs affect the development of DM complicated with cancer have only started to be revealed in recent years. Insulin-like growth factor 1 receptor (IGF-1R) signaling is a master regulator of pathophysiological processes directing DM and cancer. In this review, we briefly summarize a number of well-known miRNAs and lncRNAs that regulate the IGF-1R in DM and cancer, respectively, and further discuss the potential underlying molecular pathogenesis of this disease association.

## 1. Introduction

Diabetes mellitus (DM) is a metabolic disorder characterized by hyperglycemia, which arises from insufficient insulin secretion, insulin resistance, or augmented glucagon production. The world’s two most common diseases, cancer and type II diabetes (T2DM), share many overlapping risk factors and predisposing pathological conditions, with obesity as the most prominent. This suggests that a potential connection may exist between them. Recent epidemiological and clinical studies have shown a direct association between the incidence of diabetes and various types of malignancies, particularly hormone-associated cancer originating from pancreatic, breast, endometrial, or colorectal epithelium [1,2,3,4]. Meta-analyses of both case-control and prospective cohort studies further confirmed that T2DM supports the development of these cancers with risk ratios ranging from 1.20 (95% CI, 1.11–1.30) for breast cancer to 2.6 (95% CI, 1.6–4.1) for pancreatic cancer [5,6,7,8]. Some pancreatic cancer patients with DM were found to have a shorter history of diabetes (generally < 2 years) at the moment of cancer diagnosis, indicating that diabetes may not be the pre-existing disease, but that it may in fact be caused by pancreatic cancer [9]. However, this situation has been recorded only in pancreatic cancer and may be explained by the fact that both DM and pancreatic cancer alter the biological functions of the same organ, the pancreas.

There are also cancer types for which epidemiological studies have revealed an inverse correlation for T2DM co-morbidity. For instance, a pooled analysis of 44 studies showed that T2DM reduces the risk of developing prostate cancer by 17%, suggesting that the interaction between diabetes and cancer is multifactorial and can be highly dependent on the tissue type as well as on the factors initiating the malignant transformation [10]. Nevertheless, based on experimental, clinical, and epidemiological data, cancer is now considered as one of the most severe complications of T2DM.

Although the T2DM-cancer epidemiological relationship has been known for many decades, the exact mechanisms linking these conditions are yet to be fully understood. Considering obesity is a cause of the vast majority of T2DM diabetes, one underlying pathogenic mechanism could involve inflammation within adipose tissue with production of cytokines that interfere with insulin signaling, leading to resistance to insulin and decreased glucose clearing. The latter stimulates the pancreas to generate insulin, which leads to relative hyperinsulinemia [11,12]. Thus, insulin and insulin-like growth factor type 1 (IGF-1) signaling pathways represent master regulators of pathophysiological processes directing obesity, DM, and cancer.

Non-coding RNAs (ncRNAs) refer to a large category of RNAs that commonly do not have protein encoding ability, but have a wide repertoire of biological functions through regulation of protein expression and functions [13]. Among the different species of ncRNAs, the two most investigated classes are microRNAs (miRNAs) and the long non-coding RNAs (lncRNAs). The clustered miRNAs, miR-15a/16-1, located at chromosome position 13q14, are the first miRNAs identified in abnormally expressed in cancer (specifically down-regulated in B-cell chronic lymphocytic leukemia) [14,15]. Since then, studies on the roles of miRNAs and lncRNAs in cancer biology have burst into the scene. Specific expression of various ncRNAs associated with tumor phenotypes have been identified, indicating their importance as predictive biomarkers for cancer diagnosis and prognosis, as well as prospective therapeutic targets [16,17,18,19]. LncRNAs also impact gene regulatory networks and influence the essential pathological processes of cancer, such as metabolism [20,21]. The insulin receptor (IR) and the IGF-1R, prevalently dysregulated signaling pathways in cancer and DM, have been reported to regulate or be regulated at different levels by ncRNAs [13]. This integrative loop finely tunes multiple biological processes. By reviewing current literature, we aim to discuss the interaction between ncRNAs and the IGF-1R in the regulation of the carcinogenic process in patients with DM. 

## 2. Role of IGF-1R Signaling in the Pathogenesis of DM and Cancer

The insulin and IGF family includes ligands, receptors, and proteins that modulate ligand bioavailability, also known as IGF-binding-proteins (IGFBPs). Currently, there are three recognized ligands (insulin, IGF-I, and IGF-II), along with three cell surface receptors, including the insulin receptor (IR), IGF-1R, and IGF-2R, and seven IGFBPs [22,23]. During evolution in mammals, the IR gene has acquired an additional exon 11 that can be alternatively spliced generating two distinct isoforms: the full-length (exon 11+) IR-B, well known for its major role in controlling the glucose uptake and the shortest (exon 11−) IR-A also known as the “fetal” isoform for its recognized growth and anti-apoptotic roles during embryonic development [22,24]. Nevertheless, the IR-A is also expressed in various adult tissues under physiological conditions (e.g., brain) or pathological circumstances (e.g., insulin-target tissues in type 2 diabetes or cancer cells) [22].

The complexity of the system is further increased by the fact that cells expressing both IGF-1R and IR could generate IGF1R/IR hybrids, containing a half-IGF-1R linked to an insulin half-receptor. IGF-1R is activated mainly by IGF-I and IGF-II, while both IR-isoforms display almost the same high affinity for insulin [25]. Yet, IR-A has a high affinity for IGF-II and low affinity for IGF-I, with the IR-B displaying low affinity for the IGF-II and much lower for the IGF-1. Therefore, hybrid receptors have different affinities for IGF-I, IGF-II, or insulin depending on whether the IR-B or IR-A is recruited to such hybrid receptors (for an extensive review of various ligand-receptor affinities please see [25]).

In addition to these classical members of the IGF-1R family, several other ligands (i.e., the antimicrobial peptide LL-37) [26], and receptors (i.e., the orphan insulin-receptor-related receptor and the IR/IGF-1R hybrid receptor), could also be considered as members of the IGF-1R family [27].

**Figure 1:**
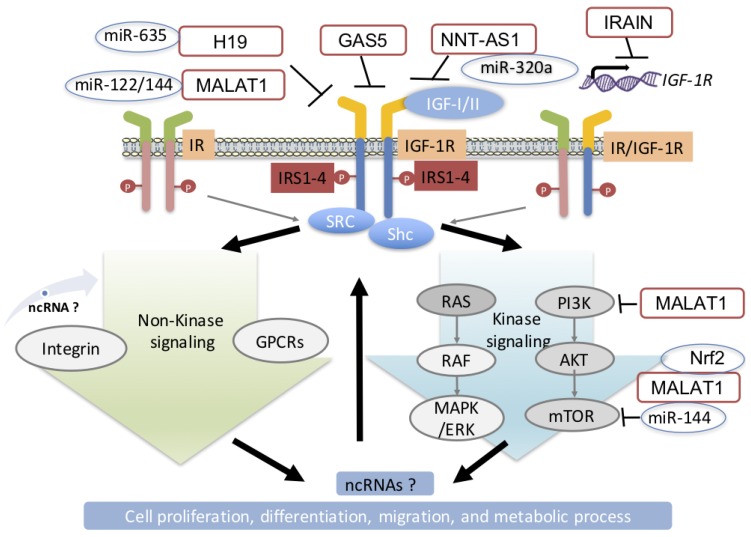
Insulin-like growth factor (IGF)-insulin receptor (IR) signaling and the regulating of insulin-like growth factor 1 receptor (IGF-1R) by long non-coding RNAs (lncRNA). The activation of IGF-1R, IR or hybrid IR/IGF-1R leads to the activation of two main downstream pathways: 1) RAS-RAF-MAPK/ERK; 2) PI3K-AKT-mTOR, to regulate metabolic processes and tumor growth; the lncRNA inhibits the IGF-1R either directly, or by acting as a sponge for the microRNAs (miRNA) interactors with IGF-1R, or as a platform connecting the interaction molecularly. Non-coding RNAs (ncRNAs) that regulate non-kinase signaling or are regulated by IGF-1R warrant further investigation.

The IR, IGF-1R, and IR/IGF-1R hybrids are the only receptor tyrosine kinases (RTKs) expressed as preformed dimers. Each part of the dimer is comprised of extracellular α subunits and transmembrane β subunits, with the latter including the tyrosine kinase domain within the intracellular region [27]. Binding of the ligand (IGF-I, IGF-II, or insulin) to the extracellular part of their cognate receptor leads to the autophosphorylation of three key tyrosine residues in the kinase domain, leading to increased kinase activity and subsequent phosphorylation of several other tyrosine and serine residues outside the kinase domain of IGF-1R or IR [22]. This process, also known as canonical kinase signaling (Figure 1), creates docking sites for downstream signaling molecules. These include insulin receptor substrates 1–4 (IRS1–4), and Src homology and collagen domain protein (Shc), which further activate several signaling pathways, including two of the most common dysregulated signaling pathways in cancer and DM: phosphoinositide 3-kinase (PI3K)-AKT and RAS-RAF-MAPK/ERK [22,28]. Phosphorylation of extracellular signal-regulated kinases (ERK) and protein kinase B (PKB) are critical for the control of most biological processes that are associated with DM/cancer comorbidity, including cell proliferation, migration, and survival, as well as glucose metabolism through the activation of GLUT4-dependent glucose uptake [29]. Some components of these kinase-activated pathways (e.g., mTOR complex 1, ERK1/2, and JNK) also trigger feedback control loops, by desensitizing the kinase signaling cascades [27]. The IGF-1R, as many other RTKs, is able to initiate downstream signaling completely independently of ligand-binding through means of transactivation by other plasma-membrane receptors such as other RTKs, G-protein-coupled receptors (GPCRs), or integrins [30,31]. Likewise, the IGF-1R could activate downstream non-kinase signaling pathways in a kinase-independent manner by employing the signal transduction machinery traditionally known to be used by the GPCR (e.g., the GRK/β-arrestin system and G-proteins) [32,33]. Equally important, components of these non-canonical, kinase-independent pathways orchestrate feedback mechanisms controlling the IGF-1R expression, trafficking, and signaling [34,35,36].

As critical initiators of these pathways, IGF-1R coordinates a complex downstream network and, thus plays essential roles in the regulation of cell growth, proliferation, survival, and cell motility, as well as on energy metabolism and glucose homeostasis. The role of IGF-1R in DM pathogenesis is complex, depending on different stages of DM. In the pre-diabetes stage, the interruption of IGF-1R signaling in the muscles and fat tissue, the primary tissues involved in the glucose metabolism will lead to insulin resistance and progression to DM [37,38]. However, during development of DM, IGF-1R was found overexpressed or activated in other organs and tissues in response to hyperglycemia and hyperinsulinemia, thereby causing deterioration of DM. For example, IGF-1R was found to be overexpressed in the vascular smooth muscle cells and brain of a DM mouse model, causing atherosclerosis and diabetic encephalopathy, two common DM complications [39,40]. Research has also shown that the IGF-1R ligand, IGF-II, is a key regulator of this feedback loop. Relatively elevated concentrations of insulin induced by insulin resistance boosts hepatic IGF-I synthesis and increases circulating IGF-I activity, which then also binds to IR, IGF-1R, and IR/IGF-1R to enhance the signaling [41]. In conclusion, hyperinsulinemia in DM activates IGF1-R and triggers downstream target effectors. This review focuses on the IGF-1R due to its central role in the above presented signaling network. However, the importance of IGF-II, the most abundant peptide from the IGF family in human circulation, should also be highlighted, and was comprehensively reviewed by Holly et al. within this special focus issue [42].

IGF-1R signaling also has crosstalk with molecules involved in inflammation, which is also a common biological process of DM and cancer. The overproduction of inflammation cytokines, in particular tumor necrosis factor-α (TNF-α) and interleukin-6, have been found to be associated with insulin resistance and the initiation and development of DM and cancer [43]. These inflammatory reactions are partly influenced by the activation of intracellular mechanisms, the kinase-β/NF-κB axis, which has also been closely linked to IGF-1R signaling by AKT and its kinase function. In lung carcinoma, overexpression of IGF-1R induces the activation of AKT/MEKK3, and then promotes the response of NF-κB signaling to TNF-α. This effect could be abolished by the inhibition of PI3K or ERK [44,45].

## 3. The Mechanism of miRNAs and lncRNAs in the Regulation of Cell Biology Processes

**Figure 2:**
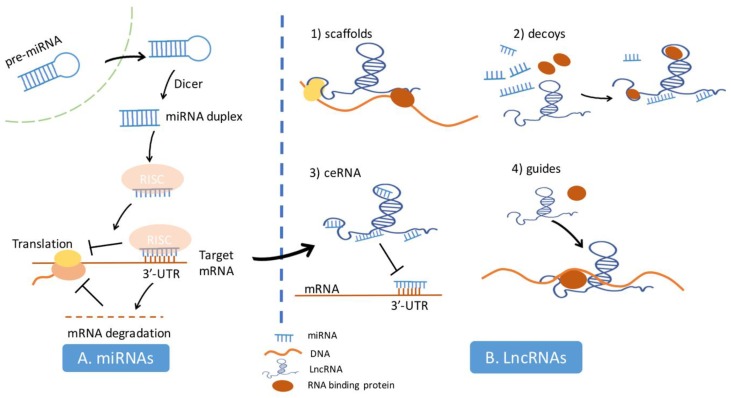
The synthesis and biological function of miRNAs and lncRNAs. **A**. Precursor miRNA (pre-miRNA) is transported from the nucleus to the cytoplasm and cleaved by Dicer to produce the miRNA duplex. One strand of the duplex would be assembled into the miRNA-induced silencing complex (RISC) with the Argonaute protein and other co-factors. By base-pairings between miRNAs and their complementary sequences in the 3’-untranslated region (3′-UTR) of target mRNA, RISCs binds to the target mRNAs and suppresses its translation or induces its degradation to regulate the down-stream biological process. **B**. lncRNA regulates the biological processes of cells through four main mechanisms as described in the main text.

MiRNAs are RNAs of approximately 21–25 nucleotides long that post-transcriptionally regulate gene expression. They originate from the cleavage of precursor miRNA (pre-miRNA) by Dicer. One strand of the miRNA duplex is assembled into the miRNA-induced silencing complex (RISC) with the Argonaute protein and other co-factors. The miRNA-RISC complex works by pairing the 3′-untranslated region (3′-UTR) onto mRNA to inhibit protein translation or induce mRNA degradation, leading to the suppression of target protein production, which functions in coordinating various metabolic mechanisms such as cell growth, differentiation, and apoptosis. Since the interaction between a miRNA and mRNA mostly relies on the first eight nucleotides, a large set of mRNAs could be the targets of the same miRNA. Unlike miRNAs, lncRNAs are a category of ncRNAs consisting of more than 200 bases. They control gene expression, protein stabilization, and enzyme activity, and consequently influence biological processes through extensive mechanisms. The four classified mechanisms include: (1) serving as scaffolds to modulate the interaction between large molecules such as DNA, RNA, and proteins; (2) acting as molecular decoys for RNA-binding proteins and small RNAs, titrating away their relative abundance; (3) functioning as competitive endogenous RNA by targeting miRNAs to suppress miRNAs function; (4) guiding RNA-binding proteins to the proper site of the targeted effector molecule. Of note, each mechanism is not mutually exclusive, but integrated with one another (Figure 2).

## 4. Modulation of IGF-1R in Diabetes and Cancer by miRNAs

**Figure 3:**
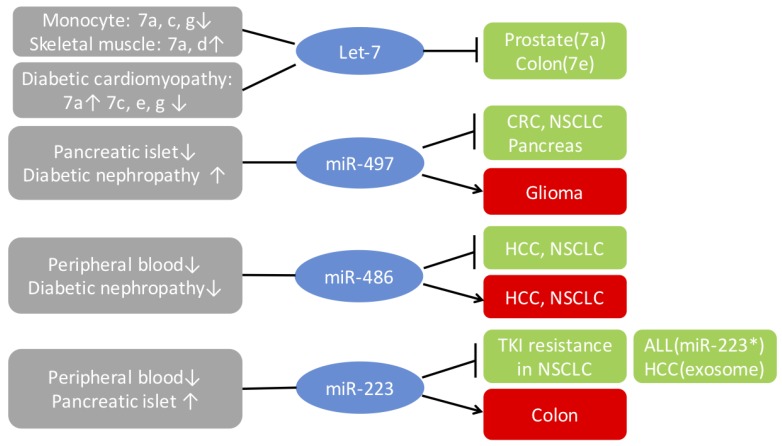
MiRNAs regulate the IGF-1R in diabetes mellitus (DM) and cancer. Green boxes highlight the suppressing role of miRNAs on the growth of specified types of cancer, inhibiting the progression of tumorigenesis. Red boxes represent the promoting effect of miRNAs on the growth of the specified kind of cancer.

MiR-375 is the first miRNA that was found to suppress glucose-induced insulin secretion and exocytosis by targeting myotrophin, thus contributing to the development of T2D [46]. Since then, dozens of additional miRNAs have been identified as components of pathways involved in the pathology of DM. This subject was discussed in numerous papers and in a meta-analysis of profiling research, which analyzed dysregulated miRNAs in DM by tissue and species specificity [47]. One miRNA might exert specific expression patterns or actions in different tissues, especially in liver and adipose tissues. Among the thousands of miRNAs that are involved in DM pathogenesis, some of them were identified as post-transcriptional regulators of IGF-1R signaling [48]. Here we concentrate on the most frequently reported miRNAs to linked to IGF-1R and summarize their function in DM/cancer comorbidity (Figure 3).

### 4.1. Let-7 Family

The let-7 family consists of a group of members, let-7a, b, c, d, e, f, g, i, miR-98, and miR-202, present in human and mice. These members have slight differences in sequence but similar biological functions due to a shared seed region from nucleotides 2–8 (TGAGGTAG), in all except miR-202. This “seed” region is responsible for interaction with target mRNAs to control degradation and stability of mRNA [28]. The role of let-7 in regulating metabolism was first reported in 2011, where researchers found that T2DM patients overexpressed let-7a and let-7d in skeletal muscle [49]. Mouse studies then revealed that the let-7 family controls glucose homeostasis and decreases insulin sensitivity by targeting multiple key factors including IGF-1R, IR, and IRS2. In addition, knocking down members of the let-7 family restored expression of IGF1-R and IR, reversing the impaired glucose tolerance by activating the AKT-mTOR pathways [50,51].

However, the dysregulation of the let-7 family in DM is inconsistent in different reported research papers, indicating that their biological roles could be dependent on tissue type and different stages of DM. For instance, in the insulin 2 mutant Akita mouse heart, a genetic mouse model of diabetes with cardiomyopathy, researchers discovered important down-regulation, but not up-regulation of let-7a [52]. Another study in a DM mouse model revealed that in the heart most members of the let-7 family, including let-7c, were downregulated as compared to controls [53]. These findings are compatible with the model of IGF-1R’s spatiotemporal function in DM, indicating that let-7 is involved in DM by affecting specific tissues, but once DM occurs, it acts as a barrier for the involvement of additional tissues.

Let-7 has also been shown to be implicated in a link between inflammatory factors and the development of T2DM. Monocytes are the precursors of macrophages that generate pro-inflammatory cytokines, causing low-grade inflammation that induces insulin resistance. Data acquired from miRNA expression microarrays in the monocytes of T2DM patients found that let-7a, -7c, and -7g were down-regulated compared to non-diabetic controls, along with an up-regulation of TNF-α [54]. TNF-α induces the activation of vascular soft muscle, neurons, and endothelial cells and reduces the expression of let-7’s via Lin-28 homolog B (LIN28B) and aggravates diabetic vascular disease [55]. However, in skeletal muscle, TNF-α has been reported to up-regulate both promoter activity and let-7 miRNA expression and down-regulate the IGF-1R, INSR, and IRS2 [56].

Let-7 family members are frequently downregulated in various cancers and regarded as tumor suppressors [57]. Conversely, IGF-1R, one target of let-7, has been shown to be commonly overexpressed in cancer. Let-7a down regulates the IGF-1R by targeting the 3’UTR of IGF-1R mRNA and is accompanied by suppression of Elk1 and c-fos, leading to growth inhibition of prostate cancer [58]. In colon cancer, let-7e led to a significant reduction IGF-1R protein level and downstream Akt inhibition to suppress tumor growth [59].

### 4.2. MiR-497

Similar to the let-7 family, not all reports are consistent on the role of miR-497 in DM. Increasing proof indicates that miRNAs can alter the equilibrium between insulin secretion and synthesis. MiR-497 was one of the miRNAs found to promote the secretion of insulin from pancreatic β-cells under high glucose levels by repressing mitochondrial uncoupling protein 2, which is a negative regulator of insulin secretion [60]. In addition to insulin resistance, another significant pathogenesis of DM is the failure to produce β-cell insulin in the pancreas, particularly in Type 1 DM. Thus, in this context, miR-497 is essential for maintaining the complex biological function of pancreatic β-cells to prevent DM. On the other hand, in an analysis of miRNA expression in the pancreatic islets of spontaneously diabetic rats, miR-497, together with miR-150, was significantly upregulated but not downregulated as expected, suggesting that the higher expression of miR-497 might promote the initial phase of DM [61]. In another study investigating the patients with Type 1 DM, the level of miR-497-5p increased during the first year after diagnosis of DM, but eventually decreased at 5 years after treatment, and was neither associated with HbA1c nor with activated C-peptide, indicating that it did not parallel the declined β-cell function in the early course of DM [62]. This means that the same miRNAs may play distinct functions based on the stages of the disease or different tissues affected. For instance, apart from the pancreas, in the kidney, miR-497 shows a protective role in attenuating the deterioration of diabetic nephropathy by decreasing proteinuria as a downstream effector of TGF-β [63].

Generally, in the early phase of DM, miR-497 is downregulated, as it is in several cancer types (breast, gastric, colorectal, and pancreas), suggesting that it plays a role as a tumor suppressor. Losing expression of miR-497 might lead to the initiation of tumorigenesis. In colorectal cancer (CRC), down-regulation of miR-497 caused by the DNA copy number reduction of chromosome 17p13.1 would up-regulate the IGF-1R or IRS1, and activate PI3K/Akt signaling, contributing to the survival of CRC cells by promoting tumor growth and resistance to chemotherapy [64,65]. Targeting IGF-1R is a therapeutic strategy for cancer, up regulation of miR-497 slowed pancreatic cancer progression and increased the sensitivity to gemcitabine [66]. Similar studies of cervical cancer and non-small cell lung cancer (NSCLC) models were also reported [67,68]. However, the impact of miR-497 is reversed in glioma. Upregulation of miR-497 has been found in human glioma and it enables cells to develop resistance to temozolomide treatment by targeting mTOR and Bcl-2, indicating the diverse or even oppose functions of miR-497 in cancer [69].

### 4.3. MiR-486

Impaired glucose tolerance (IGT) is a stage that occurs before the onset of diabetes. Dysregulated miRNAs between prediabetes and DM could be identified and regarded as potential biomarkers to screen people at high risk of developing diabetes. For instance, circulating miR-486-5p/miR-486 were found to be downregulated in patients with DM as compared to healthy controls [70,71]. MiR-486-5p was also downregulated in diabetes patients, as compared to individuals with IGT, suggesting it plays a role in the development of DM from prediabetes [70]. In Asian Indians, a high risk population for DM, miR-486 is a protective factor for reducing glycemic progression and is negatively associated with the development of glycemic impairment (OR 0.5 (0.3–0.8), *p* < 0.01) [71]. By using miR-486 as a single index to discriminate T2DM patients from normal controls, the area under the ROC curve was 0.698 (95 % confidence interval: 0.540–0.856), and the sensitivity and specificity were 79.2 and 60.0%, respectively [72]. In patients with kidney disease attributed to diabetes, miR-486-5p was negatively correlated with albuminuria or estimated glomerular filtration rate [73]. 

MiR-486 is also broadly dysregulated and found to be effective biomarker in the diagnosis and prognosis of various types of cancer [74]. This microRNA can act either as a tumor suppressor or oncogene even within the same type of cancer, such as NSCLC [75,76] and HCC [77,78]. In NSCLC, it directly targets p85α, IGF1, and IGF-1R, resulting in a decrease of pAKT signaling and downstream pFoxo3a activity, thus preventing proliferation, and promoting in vitro and in vivo lung cancer apoptosis [79]. These processes depend partly on an intact p53. MiR-486-5p was discovered to be the immediate regulator of IGF-1R in HCC tissues, specifically in the HCC cell line, Huh-7 [80]. In vitro, miR-486 inhibits the mTOR, STAT3, and c-Myc, the downstream mediators of PI3K/AKT/mTOR, JAK/STAT3, and RAS/RAF/MAPK signaling regulated by the IGF-1R, leading to the repression of cell viability. By considering the target effect of miR-486 on the IGF-1R in cancer, the hypothesis that miR-486 contributes to the development of cancer in patients with DM, directly or indirectly by the IGF axis, is reasonable.

### 4.4. MiR-223

MiR-223 is a highly-conserved miRNA that is a well-known regulator of immune response and inflammation. Macrophage-mediated inflammation plays a critical role in the etiology of insulin resistance. MiR-223 has been well documented to connect insulin resistance and inflammation, and controls multifactorial signals connected with F-box and WD repeat domain containing 7 (FBXW7), toll-like receptor 4 (TLR4), and STAT to alleviate insulin resistance and obesity pathogenesis [81]. In addition, mice with knocked-out miR-223 showed reduced glucose tolerance and insulin resistance, suggesting that it plays a critical role in maintaining β-cell function. In accordance with these findings, miR-223 was discovered to be downregulated in peripheral blood. Interestingly, it was found to be upregulated in the pancreatic islets of T2DM patients, which can likely be explained by a feedback mechanism caused by high blood glucose [82]. Also, miR-223 increases the expression of GLUT4 protein to improve glucose intake, so that insulin resistance is mitigated [83].

IGF-1R and its downstream PI3K/Akt pathway is another direct target of miR-223 [84]. In NSCLC, overexpressed miR-223 can partially overcome the acquired resistance to tyrosine kinase inhibitor (TKI) by inhibiting the IGF-1R/Akt/S6 signaling pathway [85]. The expression of miR-223 in chronic lymphocytic leukemia is controlled by Notch signalings; its removal stimulated IGF-1R signaling and may alter T-ALL biology by coordinating with other genes [86]. Surprisingly, in the search for identification of miRNAs targeting the PI3K/Akt signaling pathway in inflammation-induced colorectal carcinogenesis, miR-223 was upregulated by chronic inflammation and thus it suppressed the PI3K/Akt signaling pathway, likely due to the protective feedback trying to suppress cancer growth [87]. MiR-223 was also one of the miRNAs that could be transferred between cells by exosome or other secreted vesicles. The transfer of macrophage endogenous miR-223 reduced the expression of IGF-1R in a HCC cell line and inhibited their development, indicating that intercellular transfer of miRNAs could act as a new defense against tumor development [88]. This also drives the hypothesis that miRNAs, such as miR-223, might act as circulating mediators that modulate the cross-talk of insulin sensitivity and glucose homeostasis between different organs.

There are also some other similar tumor suppressing miRNAs, such as miR-342-3p and -126, that exert their inhibiting roles on cancer cells growth or metastasis by regulating IGF signaling. They are down-regulated in DM, and have similar mechanisms of action on the IGF-axis as miR-223 [89,90].

## 5. Regulation of Insulin/IGF Signaling by lncRNA in DM and Cancer

Growing evidence suggests that a group of lncRNAs regulate glucose homeostasis and pathogenesis of DM, especially in diabetic complications [91,92,93]. These lncRNAs include Risa, HOTAIR, and Meg3 [94]. However, the number of lncRNAs reported to be associated with IGF-1R is smaller as compared to miRNAs, probably due to the tissue specificity of lncRNAs or the less amount of studies published to date on lncRNAs versus miRNAs. The lncRNAs, directly or indirectly associated with IGF-1R, that have impact on diabetes and cancer pathogenesis are highlighted here.

### 5.1. MALAT1

The lncRNA metastasis-associated lung adenocarcinoma transcript 1 (MALAT1) was first reported to be overexpressed in lung adenocarcinoma [95]. Dysregulation of MALAT1 also contributes to DMs and its complications. It was identified as a competing endogenous RNA (ceRNA) connecting miR-144 and mTOR, and forming a regulatory loop in T2DM, but the mechanisms and functions were not yet revealed [96]. Moreover, it interacts with Nrf2 and transcriptionally suppresses its expression, thus promoting ROS production, leading to the impairment of the IRS/PI3K/Akt pathway to induce insulin resistance. In MALAT1 null mice, ROS was greatly reduced in hepatocytes and pancreatic islet cells, and it was correlated with enhanced insulin signaling in response to glucose tolerance challenges [97].

In endothelial cells, under glucose excess as seen in DM, MALAT1 is reactively overexpressed. It increases the levels of inflammatory cytokines, IL-6 and TNF-α, key pathogens of diabetic micro or macrovascular complications, by activating serum amyloid antigen 3 [98]. Moreover, it has been demonstrated that knocking-down MALAT1 would alleviate retinal vessel impairment and inflammation in diabetic rats by involving the p38 MAPK signaling pathway, suggesting that it serves as a regulator of diabetic retinopathy [99].

### 5.2. GAS5

Growth arrest-specific 5 (GAS5) regulates cell proliferation, apoptosis, and invasion in numerous cancers. Recent literature implies that the pathophysiological mechanism of DM is affected by GAS5 dysregulation. Carter et al. found that the circulating level of GAS5 is negatively associated with T2DM incidence [100]. ROC curve analysis revealed that the optimal cut-off value to distinguish DM from healthy control is ≤10 ng/μL, which would achieve a sensitivity of 85.1% and specificity of 67.3%, proving that circulating GAS5 is a good candidate for screening for diabetes. However, inconsistent results were found in another study focusing on lncRNAs from peripheral blood mononuclear cells (PBMCs) in which the level of GAS5 was upregulated, along with HOTAIR, MALAT1, and X-inactive specific transcript (XIST) [101]. Downregulation of GAS5 has also been implicated in polycystic ovary syndrome (PCOS), which is a disease tightly connected with insulin resistance and inflammation pathogenesis, comparable to DM. GAS5 was also found to be a good predictor for PCOS diagnosis [102]. These data suggest that it might be involved in the development of insulin resistance in PCOS, though the molecular mechanism is not clear.

GAS5 was broadly found to be reduced in various cancer types, such as breast and lung cancer [103,104]. Further studies confirmed that GAS5 was involved in endometrial carcinoma, particularly in patients diagnosed with T2DM [105]. Recently, IGF-1R has been established as a target of GAS5 and involves TKI therapeutic sensitivity regulation. Induced expression of GAS5 decreases the IGF-1R activity and overcomes gefitinib-resistance in lung adenocarcinoma [106,107]. Combination of GAS5 with gefitinib markedly reduced the levels of phospho-IGF-1R, pEGFR, and the downstream signaling proteins pAKT and pERK, thus inhibiting tumor growth [108]. However, it is necessary to determine the precise mechanism by which GAS5 governs the IGF-1R phosphorylation. A further study is needed to explore the effectiveness of a combination therapy that would include GAS5 in lung adenocarcinoma, particularly in patients with EGFR-TKI resistance.

### 5.3. IRAIN

The newly identified 5.4 kb IGF-1R antisense intragenic noncoding RNA (IRAIN), is a lncRNA which is translated by an intronic IGF-1R locus promoter. It is downregulated in various types of cancers, functioning as a putative tumor suppressor. By interacting with both IGF-1R and the intronic DNA enhancer (by intrachromosomal loop), IRAIN is preventing cancer development through declined IGF-1R expression [109,110]. With the rebalance of the IGF-1R/IRAIN ratio by depletion of IRAIN using the CRISPR system, breast cancer cells exhibited a significant reduction in proliferation, migration, and invasion capacities [111]. No study has yet reported the roles of IRAIN in DM, making it a potential area for further research.

### 5.4. Other lncRNAs that Regulate the IGF-1R

Other lncRNAs regulate the IGF-1R indirectly by acting as a platform controlling the molecular interactions. For example, the IGF-1R inhibition by miR-320a is released by the nucleotide nicotinamide transhydrogenase antisense RNA 1 (NNT-AS1) with consequent increase in IGF-1R level due to binding of miR-320a. This activates the PI3K/AKT signaling pathway, and ultimately promotes osteosarcoma development [112]. H19 is a common oncogenic lncRNA that is found to be overexpressed in many solid tumors, though its dysregulation in DM remains conflicting. Farzi-Molan et al. found it was significantly decreased in the muscles of patients with T2DM and in insulin-resistant mice. Depletion of H19 would result in bioavailability of let-7, triggering IR removal, thereby impairing the oxygen signal and reducing sugar intake [113]. However, in another study of a DM mouse model, hepatic H19 was upregulated and contributed to hyperglycemia [114]. Since let-7a is also a regulator of IGF-1R, H19 depletion will ideally lead to a decrease in IGF-1R. However, it was found that lncRNA H19 inhibition is concomitant with IGF-1R up-regulation instead of down-regulation; this process is mediated by the miR-675 transcribed from H19 locus, suggesting that H19 regulation loops plays a pivotal role in determining the direction of regulation [115]. In summary, the relationship between H19/IGF-1R and insulin resistance is currently inconsistent in the literature, which warrants further investigation (Figure 1).

## 6. Concluding Remarks

The roles of the IR and IR/IGF-IR were reviewed, along with their association with ncRNAs in regulating DM and cancer [25,116]. We discussed a number of well-known miRNAs and lncRNAs that regulate IGF-1R signaling in DM and cancer, and further highlight the potential underlying molecular pathogenesis of their comorbidity. Hence, miRNAs and lncRNAs are regarded as attractive potential biomarkers for diagnosis and therapeutic targets in DMs and cancer through the regulation of IGF-1R. Many studies have validated their role in diagnosing and monitoring the occurrence and development of DMs and cancer, some of them being currently explored in clinical trials to evaluate the diagnostic test sensitivity and efficiency. In addition, circulating miRNAs are more stable and can be detected in plasma, therefore they have been very promising as minimally aggressive biomarkers. Overall, ncRNAs have substantial potential as biomarkers in DMs and cancer. However, ncRNAs that regulate non-kinase signaling and the ncRNAs controlled by the activated IGF-1R warrant further investigation.

The principal therapeutic approach is to restore miRNAs that target and downregulate the IGF-1R expression and/or signaling during the development of DM, which is extensively discussed in previous reviews [116,117]. Delivering miRNA directly or using exogenous recombinant viral vectors are two common therapeutic approaches under development. Although numerous in vitro and in vivo studies have proven that the strategy is feasible in the treatment of DM as well as in cancer, there are still challenging issues that need to be addressed for targeting the IGF-1R, especially in clinical settings [118,119]. As discussed above, some ncRNAs have a complicated role in regulation, which makes the choice of intervention timing complicated. Collectively, this review demonstrates that ncRNAs involved in IGF-1R signaling, directly or indirectly, plays an important role in linking insulin resistance to DM and cancer.
